# Correlation of Vascular Endothelial Growth Factor subtypes and their receptors with melanoma progression: A next-generation Tissue Microarray (ngTMA) automated analysis

**DOI:** 10.1371/journal.pone.0207019

**Published:** 2018-11-08

**Authors:** S. Morteza Seyed Jafari, Christina Wiedmer, Simone Cazzaniga, Živa Frangež, Maziar Shafighi, Helmut Beltraminelli, Benedikt Weber, Hans-Uwe Simon, Robert E. Hunger

**Affiliations:** 1 Department of Dermatology, Inselspital, Bern University Hospital, University of Bern, Bern, Switzerland; 2 Centro Studi GISED, Bergamo, Italy; 3 Institute of Pharmacology, University of Bern, Bern, Switzerland; 4 Department of Biomedical Research, University of Bern, Bern, Switzerland; 5 Department of Dermatology, Medical University of Vienna, Vienna, Austria; Institut national de la recherche scientifique, CANADA

## Abstract

**Introduction:**

Finding new markers to assess prognosis of melanoma without the necessity to perform a surgical interventions is an important goal in melanoma research. The current study aimed to assess the correlation of clinical course and prognosis of primary and metastatic melanoma with expression of VEGF family and their receptors.

**Methods:**

A ngTMA block was made from the randomly selected paraffin tissue blocks of the patients with melanocytic nevi, primary and metastatic melanoma. Then sections cut from ngTMA-block were immunohistochemically stained with proper antibodies. Expression of these proteins was investigated using automated image analysis and compared among the study groups.

**Results:**

We analyzed the tissue of 238 patients with following diagnoses: 101 (42.4%) with a diagnosis of nevus, 86 (36.1%) Malignant melanoma and 51 (21.4%) metastasis. Median follow-up time for the malignant lesions was 5.71 years. Among the tested antigen, VEGF-C (p = 0.016), VEGF-R2 (p<0.001) and VEGF-R3 (p = 0.002) were significantly higher expressed in the metastatic tissues. When these scores were assessed in multiple regression models, the only independent factor linked to patient’s diagnosis was VEGF-R2 (p<0.001). In addition, groups of highly correlated variables (VEGF-C and VEGF-R3, VEGF-A and VEGF-R1) were found to form separate sub-clusters. On the other side, high values of VEGF-C were associated with both overall and disease-free survival with a statically significant HR of 2.76 (95% CI: 1.27, 5.98; p = 0.01) and 2.82 (95%CI: 1.62, 4.91; p<0.001), respectively.

**Conclusions:**

This study shows that VEGF-C and VEGF-R2 might represent new prognostic marker in MM. However, further prospective studies are warranted to test their real efficacy as a prognostic marker.

## Introduction

Malignant melanoma (MM) is a malignant tumor arising from an uncontrolled growth of melanocytes, [1, 2] responsible for 90% of the deaths associated with cutaneous tumors. [1, 3, 4] The prognosis of patients with melanoma is tumor-stage dependent. The most important prognostic marker is he Breslow-thickness followed by the presence of an ulceration, and the presence of mitoses. Sentinel lymph node biopsy (SLNB) is a further procedure to acquire some more prognostic informations. [5–7] However, this surgical procedure is costly and might cause significant complications such as lymphocele (23%), wound infection (19%), extremity swelling (17%), and seroma (15%). [8, 9] Thus, it is an important goal in melanoma research, to find markers to assess the metastatic risk of disease without having to perform a surgical intervention.

In tumorigenesis, the formation, differentiation and growth of blood vessels are absolute necessary for the development, expansion, and spread of a tumors. [10, 11] Moreover, the vascular network is also fundamental to allow the metastatic cascade, [12, 13] which requires the transport of malignant cells through the blood and/or lymph vessels. [14] The expression of some important angiogenic factors as the Vascular Endothelial Growth Factor (VEGF) subtypes and their receptors (VEGF-Rs), is upregulated on vascular endothelial cells during tumor angiogenesis and correlates with tumor growth rate, microvessel density, tumor proliferation, tumor metastatic potential and finally poorer patient prognosis. This has been demonstrated in different malignant tumours and in different organs [11, 14–16]: breast, [17] colon, [18, 19] lung, [20, 21] thyroid, [22–24] gastric [25] squamous cell cancers, [26] mesotheliomas, [27] neuroblastomas, [28] and sarcomas. [26]

However, the role of such angiogenic factors on the clinical follow up and disease-prognosis, inclusive patient-survival in MM remains unclear. [13] With this study for the first time, we aimed to assess the correlation between the expression of various VEGF/receptors, and the clinical course and prognosis of patients with primary and metastatic melanoma.

## Material and methods

### Study population and clinical data collection

The analyses have been performed on the paraffin tissue blocks from the archives of the Dermatopathology Unit at the Department of Dermatology, Inselspital, Bern University Hospital, Switzerland. (2003–2015) We randomly selected cases with following diagnosis: melanocytic nevi (n = 120), primary melanoma (n = 134) and metastatic melanoma (n = 76). The blocks with proper and enough tissue for the immunohistochemical staining (minimum of 4 mm thickness) were included in the study. Furthermore, the patients without complete documents and/or regular clinical follow-up were excluded. This study was conducted in accordance with the standards of the Ethical Committee of the Canton of Bern on human experimentation and with the Helsinki Declaration of 1975, as revised in 1983.

We collected data of patients’ gender, age, tumor anatomical location, tumor type, Breslow thickness, ulceration, sentinel lymph node status, presence of distant and/or locoregional metastases. In the current study, local recurrences can represent either persistent disease due to inadequate initial excision or true recurrence adjacent to the scar after adequate prior wide local excision and usually have an in situ component, or they may represent satellite metastases. Locoregional recurrence of melanoma after initial resection was defined as recurrence at the site of the primary lesion, regionally in the draining lymph node basin, or anywhere in between. [29, 30] The spreading from the original (primary) tumor to distant organs or distant lymph nodes is considered as distant metastases. [30, 31]

### Next-generation Tissue Microarray (ngTMA)

As discussed before, [32] the last histological Haematoxylin and Eosin (H&E) slide of each patient was retrieved. The H&E stained slides were scanned using panoramic Digital Slide Scanner (3DHISTECH). Using the free digital slide viewer software, the digital slides were evaluated and areas of interest for integration into the ngTMA were found. The annotation (600 μm) was thereafter moved to the desired histological structures for incorporation into the ngTMA. Then, a list of all cases with their corresponding annotations was created. The corresponding paraffin tissue blocks for all annotated digital slides were retrieved and sorted in the desired order for tissue microarraying. Next, the donor blocks were loaded up into the tissue microarrayer. Then the tissue microarrayer started to drill holes of 0.6 mm in diameter in the recipient block at the selected starting point. In the next step, using the punching tool, the instrument punched holes into the tissue from the selected donor block at the exact annotated and confirmed region. Cores (only one per patient) are then transferred from the donor to the recipient block. [32] (S1 Fig).

### Immunohistochemical staining

From the prepared ngTMA-block new sections were cut to perform immunohistochemical stainings.

The automated staining was performed using the BOND-III fully automated IHC and ISH stainer (Leica Biosystems) according to the manufacturer’s instructions. In brief, paraffin-embedded tissue sections were first dewaxed and rehydrated, followed by epitope retrievel (epitrope-retrieval solution 2; Leica). They were then incubated with following primary antibodies for 15min: Anti-VEGF-A antibody (abcam), anti-VEGF-B antibody (R&D systems), anti-VEGF-C antibody (biorbyt), anti-VEGF-D antibody (R&D systems), anti-VEGF-R1 antibody (biorbyt), anti-VEGF-R2 antibody (R&D systems) and anti-VEGF-R3 antibody (R&D systems). This step was then followed by a post-primary-IgG-linker and a Poly-AP-IgG reagent (Bond Polymer Refine Red Detection System, Leica). Sections were then developed in Fast Red substrate chromogen (Leica) (S2 Fig, S1 Table).

### Image analysis procedure

**Fig 1:**
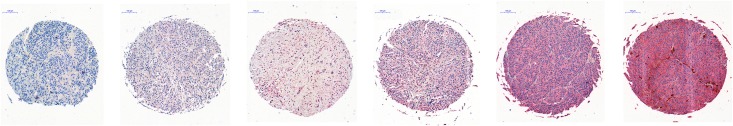
Semi-quantitative scale, as followings: (from left to right) 0 = absent; 1 = very low expression; 2 = low expression; 3 = moderate expression; 4 = strong expression; 5 = very strong expression.

The stained slides were scanned by panoramic Digital Slide Scanner (3DHISTECH). The scanned images were opened in the QuPath (Queen’s University Belfast) digital image analysis system. Then the tissue cores were automatically detected using ngTMA dearrayer. After modifications of the individual selected cores, unsuitable cores for analysis, were marked as “Missing data” and excluded. To perform automatic quantification of immunohistochemistry red stained tissue by measurement of optical density of red color—which is proportional to the expression extent of specific antigens, [33] Image J (NIH, Bethesda, MD, USA) macro runner was applied in QuPath (Queen’s University Belfast) to run the proper ImageJ macro (https://imagej.nih.gov/ij/docs/examples/stained-sections/index.html) based on extracting image regions from each ngTMA. In order to validate the full-automated image analysis, all 2975 stained cores were also evaluated by an experienced staff using a semi-quantitative scale, as followings: 0 = absent; 1 = very low expression; 2 = low expression; 3 = moderate expression; 4 = strong expression; 5 = very strong expression (Fig 1).

### Statistical analysis

Data were presented as means with standard deviations (SD) or numbers with percentages for continuous and categorical variables respectively. One-way MANOVA was used to analyse differences in the distribution of the scores across groups of patients with a different diagnosis (nevus, MM or metastasis). [34] Differences were computed along with their 95% confidence intervals (CI) and p-values. In addition, in order to assess which factors were independent predictors for the diagnosis, all variables with a p-value <0.15 in MANOVA analysis were evaluated for inclusion in multinomial logistic regression models with stepwise forward selection algorithm.

The association between each pair of scores was also investigated by means of Pearson’s *r* correlation coefficient. Groups of correlated scores were then analysed by using hierarchical clustering with Pearson’s correlation as proximity measure and centroid linkage as partitioning criterion. [35] Clusters were displayed by using a tree diagram (dendrogram), where at each node a two sub-branch of clustered group of variables are represented; the height of nodes represents the distance between pair of clusters. In addition, principal component analysis (PCA) was used as an alternative approach to show clustering among correlated variables. [36] Differences between patients with MM and metastases across demographics and clinical characteristics were tested by means of Pearson’s χ^2^ test or Mann-Whitney U test for categorical and continuous variables respectively. For analysis purposes continuous variables were also categorized by using clinically relevant cut-off points.

For each kind of diagnosis (MM or metastasis), one-way MANOVA was used to analyse differences in the distribution of the scores across demographics and clinical characteristics. All variables with a p-value <0.15 in MANOVA analysis were evaluated for inclusion in multivariate generalized linear models (GLM) with stepwise forward selection algorithm.

Overall and disease-free cumulative survival rates along with their 95% CI were computed using Kaplan-Meier estimator. For the aims of this analysis, scores were also categorized in low vs. high values based on significance of correlation with survival outcomes. [37] Optimal cut-off values were defined as the points with the most significant split according to the log-rank test, which was used to assess overall and disease-free survival differences between categorized scores.

Scores with a p-value <0.15 in the univariate survival analysis were then evaluated for inclusion in multiple Cox regression models with forward stepwise selection algorithm. The relative risk of mortality was expressed in terms of hazard ratio (HR) along with its 95% CI and p-value.

The Benjamini–Hochberg procedure was used to account for multiple comparisons in all multivariate tests by taking a false discovery rate of 0.05. [38] The critical p-values derived from the procedure were reported as reference value to determine statistically significant findings. For the purpose of validating the relationship between automated and non-automated image analysis results, Pearson normalized correlation coefficient was applied. Analyses were carried out with SPSS software v.20.0 (IBM Corp, Armonk, NY).

## Results

Complete data on study scores were obtained for 238 subjects: 101 (42.4%) melanocytic nevus, 86 (36.1%) MM and 51 (21.4%) metastasis. The median follow-up time for the malignant lesions was 5.71 years. Pearson normalized correlation coefficient showed significant strong positive correlation between automated score and semi-quantitative evaluation (non-automated score). (p<0.001, r = 0.802)

**Fig 2:**
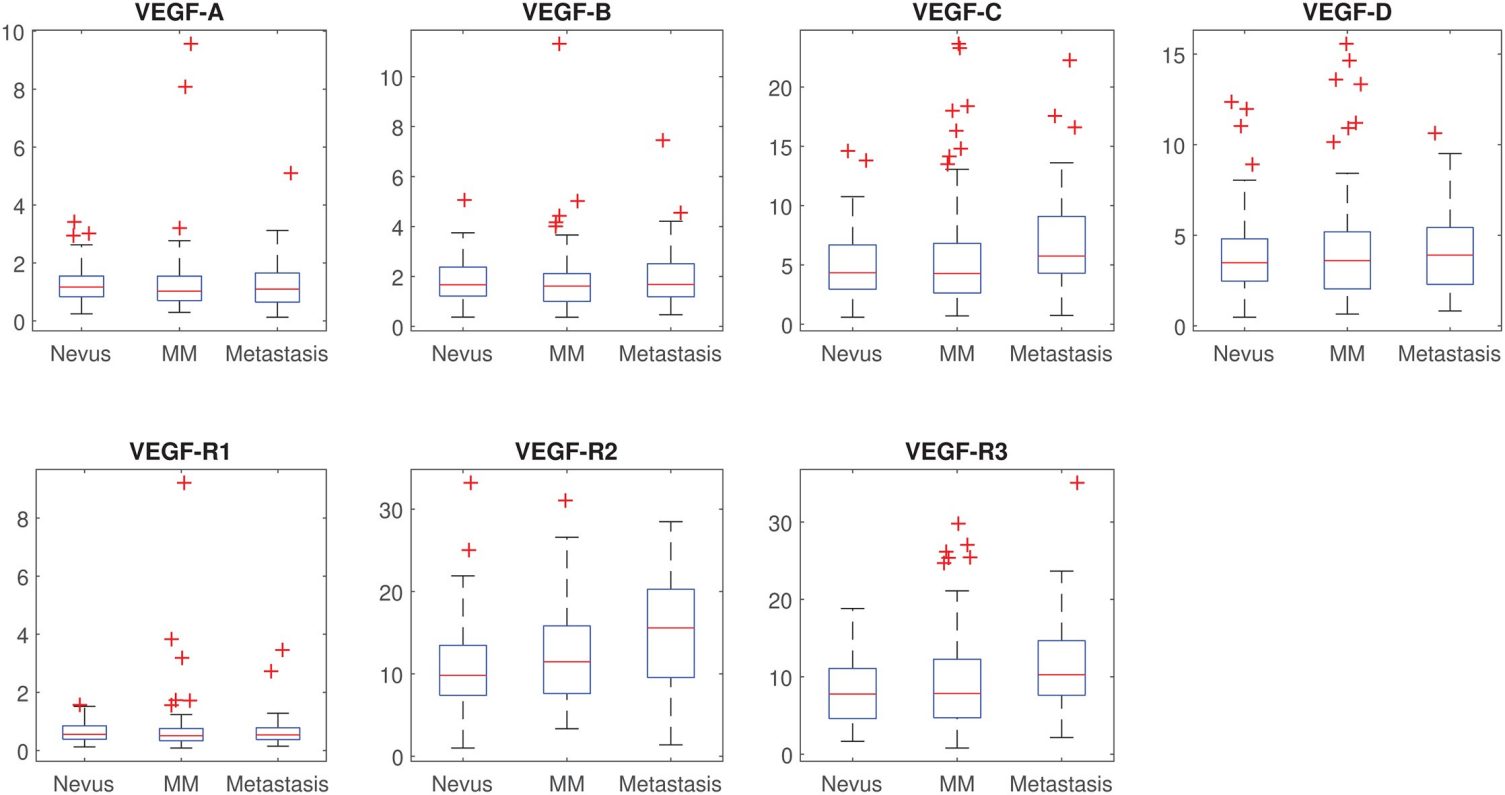
Box and whisker plots of scores by patient’s diagnosis.

Scores statistics, overall and by patient’s diagnosis, are shown in Table 1 (Fig 2). Overall there was a significant difference of the scores across diagnoses (p<0.001). More specifically the only scores significantly associated with patient’s diagnosis, after accounting for multiple comparisons, were VEGF-C (p = 0.016), VEGF-R2 (p<0.001) and VEGF-R3 (p = 0.002). For all these scores, only the difference between nevus and metastasis was statistically significant after accounting for multiple comparisons. When these scores were included in multinomial logistic regression models, the only independent factor linked to patient’s diagnosis was VEGF-R2 (p<0.001).

**Table 1:** Scores statistics, overall and by patient’s diagnosis.

		Mean, SD	Difference (95% CI)	P*, Cr. P**
VEGF-A	Nevus	1.26, 0.64	Ref.	0.868, 0.050
	MM	1.31, 1.31	0.05 (-0.23, 0.33)	0.723, 0.039
	Metastasis	1.22, 0.86	-0.04 (-0.37, 0.29)	0.821, 0.043
VEGF-B	Nevus	1.82, 0.93	Ref.	0.841, 0.043
	MM	1.83, 1.43	0.004 (-0.34, 0.35)	0.980, 0.050
	Metastasis	1.94, 1.22	0.11 (-0.29, 0.52)	0.583, 0.032
VEGF-C	Nevus	4.91, 2.76	Ref.	**0.016,** 0.021
	MM	5.73, 4.83	0.82 (-0.33, 1.97)	0.160, 0.021
	Metastasis	6.87, 4.36	**1.96 (0.62, 3.30)**	**0.004,** 0.011
VEGF-D	Nevus	3.91, 2.28	Ref.	0.697, 0.036
	MM	4.24, 3.17	0.33 (-0.43, 1.09)	0.396, 0.029
	Metastasis	4.08, 2.31	0.17 (-0.73, 1.06)	0.715, 0.036
VEGF-R1	Nevus	0.64, 0.35	Ref.	0.661, 0.029
	MM	0.73, 1.08	0.10 (-0.12, 0.31)	0.377, 0.025
	Metastasis	0.66, 0.58	0.02 (-0.23, 0.27)	0.882, 0.046
VEGF-R2	Nevus	10.75, 4.90	Ref.	**<0.001,** 0.007
	MM	12.84, 6.31	2.09 (0.39, 3.78)	0.016, 0.014
	Metastasis	15.10, 6.80	**4.35 (2.36, 6.33)**	**<0.001,** 0.004
VEGF-R3	Nevus	8.17, 4.17	Ref.	**0.002,** 0.014
	MM	9.35, 6.64	1.18 (-0.45, 2.81)	0.156, 0.018
	Metastasis	11.59, 6.31	**3.42 (1.51, 5.33)**	**0.001,** 0.007

CI: confidence interval, MM: malignant melanoma, P: p-value, SD: standard deviation

* P-values in italics are single p-values from one-way MANOVA analysis. Other p-values test the differences against a reference category for each score. The overall difference of the scores across different diagnoses is significant at p<0.001.

** Benjamini–Hochberg critical values for multiple comparisons with a false discovery rate of 0.05. A first set of tests was performed on the overall difference for each score; then a second sub-set of tests was done on the specific differences within each score. Results must be considered statistically significant (boldfaced) only when their p-values are lower than the corresponding critical values (Cr. P).

**Fig 3:**
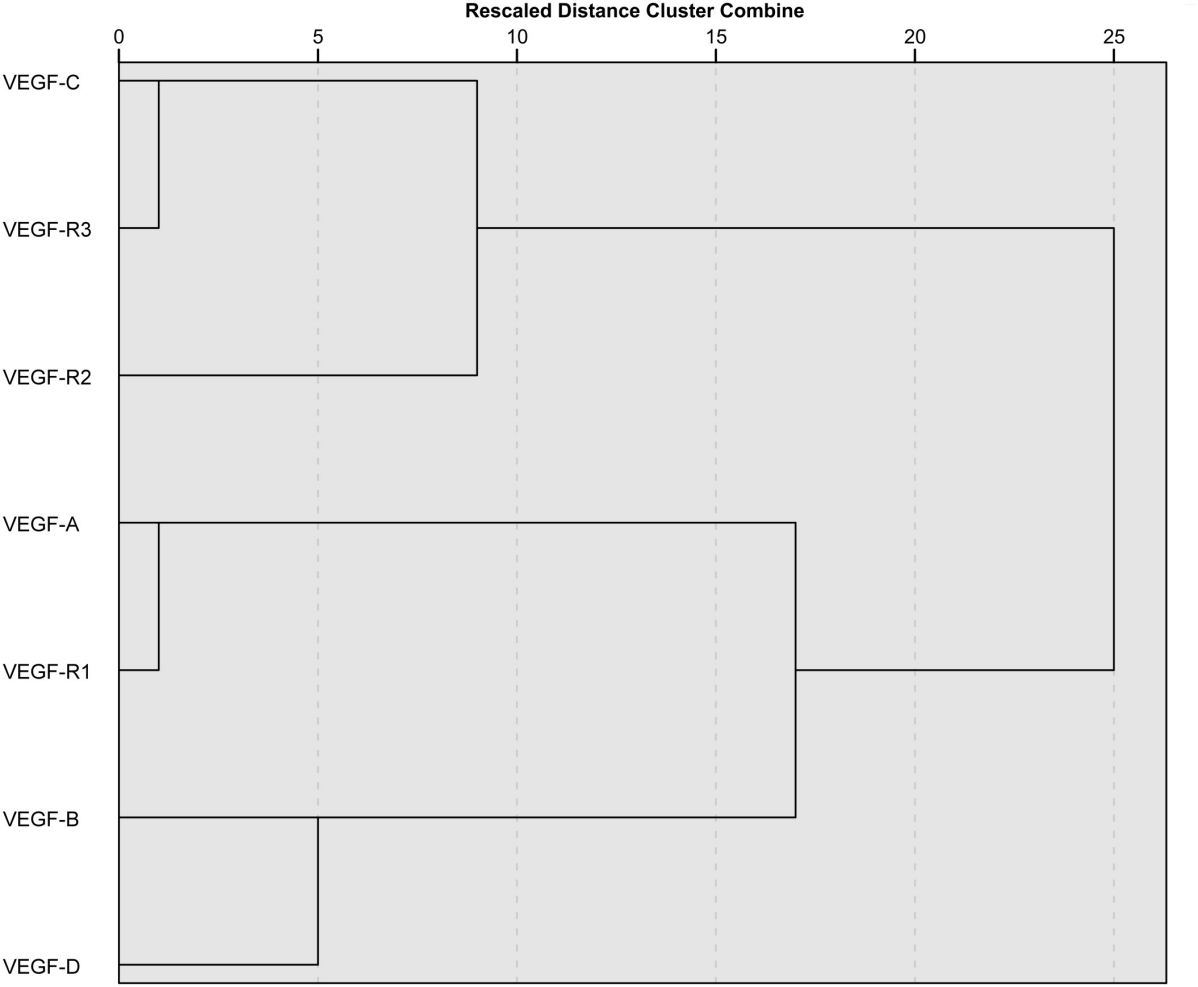
Dendrogram resulting from hierachical clustering analysis and presenting different clusters of correlated scores.

Then we explored the associations among automated scores. Overall there was a high correlation between them, ranging from a minimum of r = 0.32 for VEGF-R1 and VEGF-R2 to a maximum of r = 0.88 for VEGF-C and VEGF-R3 (S3 Table). Groups of correlated scores were also analysed using hierarchical clustering. Looking at the dendrogram (Fig 3), proceeding from right to left, it is possible to observe two large separated clusters of scores. The first one comprises VEGF-C, VEGF-R2 and VEGF-R3, the same variables significantly associated to patient’s diagnosis in MANOVA analysis, while on the other side we found the remaining variables. Going deeper down the tree, we find groups of highly correlated variables (VEGF-C and VEGF-R3, VEGF-A and VEGF-R1) forming separate sub-clusters. VEGF-D is only moderately correlated to VEGF-B. These two variables are quite distant in the graph to all the other scores. These findings were also confirmed by adopting principal component analysis (PCA) as an alternative approach (S3 Fig).

Demographics and clinical characteristics of patients with a specific diagnosis of MM or metastasis are shown in Table 2. Overall 60% of patients were males with a mean age of 62.3 ± 15.8 (mean ± SD) years and without any significant differences between the two groups. 47.0% of patients had a superficial spreading MM and 29.1% a nodular MM, with a significant different distribution between groups. 41.4% of subjects had a primary tumor located at the trunk, while 36.8% at upper or lower extremities. Ulceration was detected in 25.2% of patients with a significantly higher prevalence in the metastasis group. The average Breslow’s thickness was 2.3 ± 2.7 mm and it was higher in the metastasis compared to the melanoma group (4.2 vs. 1.4, p<0.001). A positive sentinel lymph node was found in 25.3% of the patients.

**Table 2:** Demographics and clinical characteristics of patients with a diagnosis of MM or metastasis, by kind of diagnosis and overall.

		MM = 86	Metastasis = 51	Total = 137	P**
		N*,%	N*,%	N*,%	
Sex	F	36,42.4%	18,36.0%	54,40.0%	0.467
	M	49,57.6%	32,64.0%	81,60.0%	
Age (yrs)	*Mean (SD)*	*60*.*6(16*.*0)*	*65*.*2(15*.*1)*	*62*.*3(15*.*8)*	0.091
	< 50	22,25.6%	8,16.0%	30,22.1%	
	50–64	28,32.6%	11,22.0%	39,28.7%	
	65+	36,41.9%	31,62.0%	67,49.3%	
Tumour type	NM	16,18.6%	23,47.9%	39,29.1%	<0.001
	SMM	53,61.6%	10,20.8%	63,47.0%	
	Other	17,19.8%	15,31.2%	32,23.9%	
Tumour location	Head and Neck	12,14.3%	17,34.7%	29,21.8%	0.004
	Trunk	44,52.4%	11,22.4%	55,41.4%	
	Upper Extremity	11,13.1%	7,14.3%	18,13.5%	
	Lower Extremity	17,20.2%	14,28.6%	31,23.3%	
Ulceration	No	72,86.7%	17,47.2%	89,74.8%	<0.001
	Yes	11,13.3%	19,52.8%	30,25.2%	
Thickness (mm)	*Mean*, *SD*	*1*.*4(1*.*5)*	*4*.*2(3*.*6)*	*2*.*3(2*.*7)*	<0.001
	Thin	48,55.8%	3,7.0%	51,39.5%	
	Intermediate	34,39.5%	25,58.1%	59,45.7%	
	Thick	4,4.7%	15,34.9%	19,14.7%	
SLNB findings	Negative	40,81.6%	16,61.5%	56,74.7%	0.057
	Positive	9,18.4%	10,38.5%	19,25.3%	

NM: Nodular melanoma, MM: malignant melanoma, P: p-value, SD: standard deviation, SLN: Sentinel lymph node biopsy, SSM: Superficial spreading melanoma

* Numbers may not add up to the total due to missing data

** Pearson’s X^2^ test or Mann-Whitney U test were used for categorical and continuous variables respectively

The distribution of study scores across demographics and clinical characteristics of patients with a diagnosis of MM is reported in S3 Table. Factors with a p-value<0.15 in MANOVA analysis and evaluated for inclusion in multivariate GLM were: tumor type, Breslow’s thickness and sentinel lymph node findings. The final selected factors were tumor type and Breslow’s thickness (Table 3). More specifically, VEGF-C and VEGF-R3 values significantly decreased when superficial spreading MM and other diagnosis were compared with nodular MM. On the other hand, VEGF-A, VEGF-B, VEGF-C, VEGF-R2 and VEGF-R3 values increased when intermediate Breslow’s thickness is compared to the thin one. The same did not hold for thick tumor type, where score values were not significantly different compared to thin Breslow’s group.

**Table 3:** Factors associated to study scores and selected by multivariate analysis, in patients with a diagnosis of MM.

		VEGFA		VEGFB		VEGFC		VEGFD		VEGFR1		VEGFR2		VEGFR3		P**
		Diff. (95% CI)	P* (Cr P^)	Diff. (95% CI)	P* (Cr P^)	Diff. (95% CI)	P* (Cr P^)	Diff. (95% CI)	P* (Cr P^)	Diff. (95% CI)	P* (Cr P^)	Diff. (95% CI)	P* (Cr P^)	Diff. (95% CI)	P* (Cr P^)	
Tumour type	NM	Ref.	0.563(0.046)	Ref.	0.322(0.043)	Ref.	**0.003**(0.021)	Ref.	0.066 (0.036)	Ref.	0.571(0.050)	Ref.	0.130(0.039)	Ref.	**0.023**(0.025)	**0.001**
	SSM	0.17(-0.63, 0.98)	0.666(0.039)	-0.01(-0.85, 0.83)	0.980(0.050)	**-4.41(-6.91, -1.90)**	**0.001**(0.007)	-1.99(-3.69, -.29)	0.022(0.020)	-0.07(-0.74, .59)	0.827(0.045)	-3.47 (-6.96, .02)	0.052(0.023)	**-4.78(-8.34, -1.22)**	**0.009**(0.014)	
	Other	0.48(-0.45, 1.40)	0.310(0.034)	0.54(-0.43, 1.51)	0.272(0.030)	**-4.19(-7.10, -1.29)**	**0.005**(0.013)	-1.85(-3.82, 0.12)	0.065(0.025)	0.24(-0.53, 1.01)	0.535(0.036)	-3.50 (-7.54, 0.54)	0.089(0.027)	**-5.03(-9.15, -.91)**	**0.017**(0.018)	
Thickness	Thin	Ref.	**0.028**(0.029)	Ref.	**0.002**(0.018)	Ref.	**<0.001**(0.004)	Ref.	**<0.001**(0.007)	Ref.	0.052(0.032)	Ref.	**0.001**(0.011)	Ref.	**0.001**(0.014)	**0.03**
	Intermediate	**0.78(0.16, 1.40)**	**0.014**(0.016)	**1.14(0.50, 1.79)**	**0.001**(0.009)	**3.53(1.59, 5.46)**	**0.001**(0.011)	**2.58(1.27, 3.89)**	**<0.001**(0.002)	0.58(0.07, 1.10)	0.026(0.021)	**5.06 (2.36, 7.76)**	**<0.001** (0.004)	**5.15(2.40, 7.90)**	**<0.001**(0.005)	
	Thick	0.27(-1.65, 1.10)	0.691(0.041)	0.03(-1.40, 1.47)	0.962(0.046)	-2.27(-6.56, 2.03)	0.297(0.032)	-1.86 (-4.77, 1.05)	0.207(0.029)	-0.21(-1.35, 0.93)	0.713(0.043)	-.13 (-6.11, 5.84)	0.964(0.048)	-1.45(-7.54, 4.65)	0.638(0.038)	
Primary tumour location	Head and Neck	Ref.	0.04(0.029)	Ref.	**0.007**(0.014)	Ref.	0.1(0.036)	Ref.	**<0.001**(0.007)	Ref.	0.10(0.043)	Ref.	0.026(0.021)	Ref.	0.18(0.05)	0.005
	Trunk	0.32 (-0.31, 0.95)	0.315(0.029)	0.44 (-.43, 1.30)	0.315(0.031)	2.30 (-1.03, 5.64)	0.171(0.024)	**2.12 (.63, 3.62)**	**0.006**(0.007)	0.08 (-0.35, 0.52)	0.701(0.04)	6.36 (1.37, 11.35)	0.014(0.014)	3.22 (-1.67, 8.11)	0.192(0.026)	
	Upper Ext.	.001 (-0.73, 0.73)	0.998(0.05)	0.02 (-0.98, 1.03)	0.966(0.045)	1.13 (-2.74, 5.00)	0.558(0.038)	-.02 (-1.76, 1.72)	0.982(0.048)	0.05 (-0.45, 0.56)	0.837(0.043)	2.47 (-3.32, 8.26)	0.394(0.036)	2.75 (-2.92, 8.43)	0.333(0.033)	
	Lower Ext.	**0.81 (0.23, 1.40)**	**0.008**(0.01)	**1.39 (0.58, 2.19)**	**0.001**(0.005)	**3.88 (.77, 6.99)**	**0.016**(0.017)	**3.17 (1.78, 4.57)**	**<0.001**(0.002)	0.48 (0.08, 0.89)	0.021(0.019)	**6.33 (1.67, 10.98)**	**0.009**(0.012)	5.02 (0.46, 9.58)	0.032(0.021)	

CI: confidence interval, NM: Nodular melanoma, MM: malignant melanoma, SSM: Superficial spreading melanoma, P: p-value

* P-values in italics are single p-values from multivariate GLM analysis. Other p-values test the differences against a reference category for each score.

** Overall p-value.

^ Benjamini–Hochberg critical values for multiple comparisons with a false discovery rate of 0.05. A first set of tests was performed on the overall difference for each score; then a second sub-set of tests was done on the specific differences within each score. Results must be considered statistically significant (boldfaced) only when their p-values are lower than the corresponding critical values (Cr. P).

S4 Table presents the association between study scores and demographics and clinical characteristics of patients with metastasis. Factors with a p-value <0.15 in MANOVA analysis were: sex, age and primary tumor location, which was the only factor retained in the multivariate analysis (Table 3). Looking at the specific effect of single scores across different tumor locations, VEGF-A, VEGF-B, VEGF-C, VEGF-D and VEGF-R2 values were significantly higher for lower extremities tumors compared to head and neck localisation. The value of the scores in other locations was not significantly different, apart from VEGF-D that was also significantly higher for the trunk.

Overall and disease-free patients’ survival after a diagnosis of MM or metastasis was then analysed until 15 years of follow-up. The overall cumulative survival at 5, 10 and 15 years was 83.2% (95% CI: 76.5, 89.9), 73.1% (63.1, 83.1) and 67.5% (53.4, 81.6) respectively. On the other hand, the disease free survival for the same years was of 63.6% (55.0, 72.2), 55.0% (44.6, 65.4) and 50.0% (36.7, 63.3).

**Fig 4:**
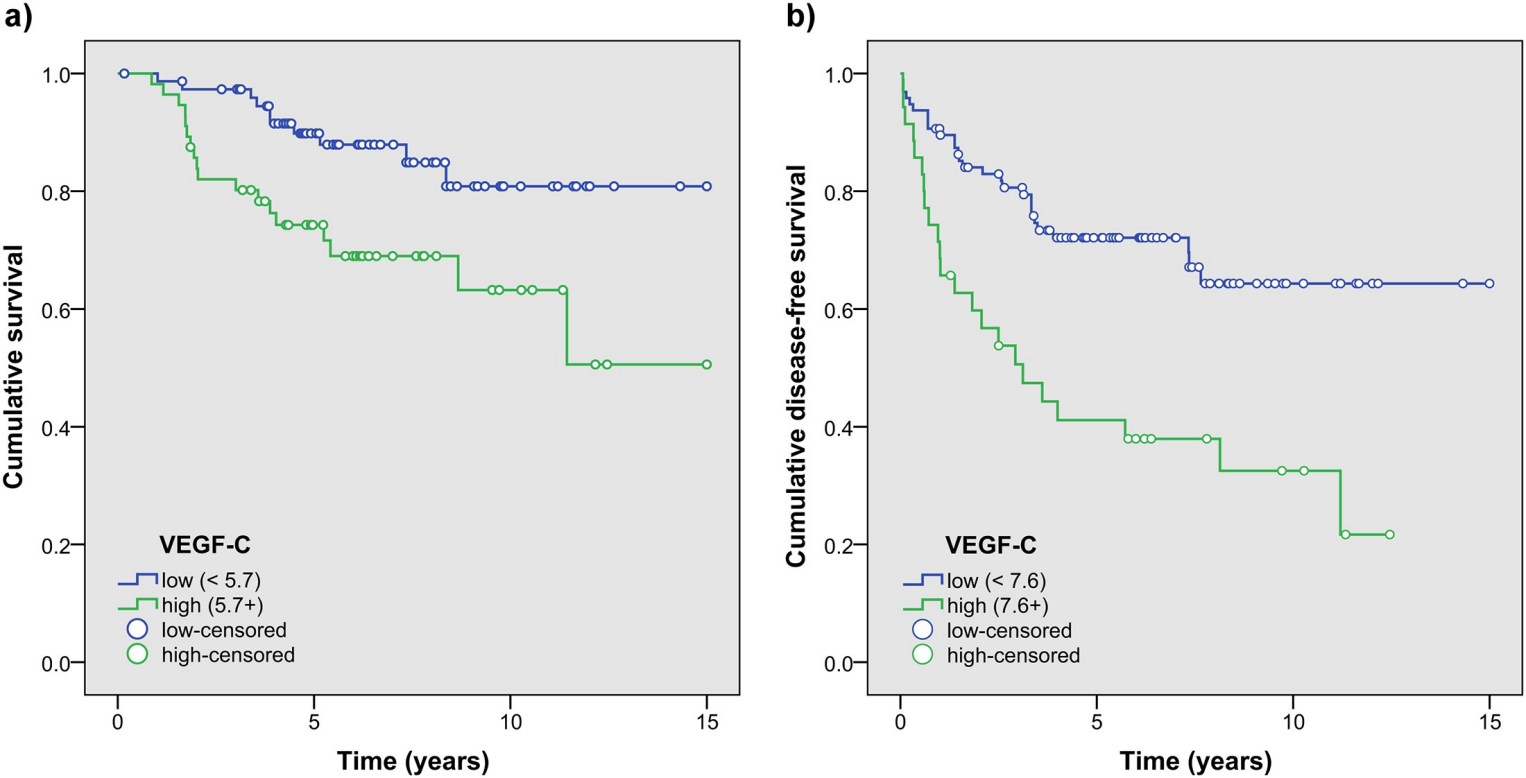
Kaplan-Meier plot of overall (a) and disease-free patients’ survival (b) between VEGF-C high vs. low values respectively, in patients with a diagnosis of MM or metastasis.

Univariate analysis of the association between optimal categorized scores and overall and disease-free patients’ survival revealed that VEGF-C, VEGF-R1, VEGF-R2 and VEGF-R3 were possible factors associated to both outcomes (S5 Table). When evaluated in Cox regression models, with stepwise selection algorithm, high values of VEGF-C were associated with both overall and disease-free survival with a statically significant HR of 2.76 (95% CI: 1.27, 5.98; p = 0.01) and 2.82 (95%CI: 1.62, 4.91; p<0.001) respectively (Fig 4, S5 Table).

## Discussion

Finding new markers to assess prognosis of melanoma without the necessity to perform a surgical interventions is an important goal in melanoma research. In this study on patients with melanoma we assessed for the first time, the correlation between clinical follow up and prognosis, and the expression of VEGF family and their receptors using a ngTMA full-automated analysis.

Malignant melanoma has been shown as an angiogenic tumor type because the vessel formation is an important step in disease progression from atypical melanocytes. However, the exact role of angiogenesis, regulation of tumor lymphangiogenesis, role of VEGFs and their receptors in the patients`survival has remained unclear. In addition, despite different studies, no antiangiogenic therapy has yet been approved for MM. [13]

Among the angiogenic growth factors, the VEGF family and their corresponding receptors play a crucial role regulating the angiogenic and lymphangiogenic processes. [11, 39] VEGF-A, VEGF-B, VEGF-C, and VEGF-D have been identified as the most important members of the family to date. [11, 40, 41] The result of current study declared, that VEGF-C and VEGF-R2 and VEGF-R3 seem to form separate sub-clusters, which might explain their interactions. Recent studies showed that, VEGF-A and VEGF-D exert their action through VEGFR-1 and VEGFR-2. While VEGF-B performs its action through VEGFR-1, and VEGF-C exerts its action through VEGFR-2 and VEGFR-3. [11, 40, 41]

Whereas VEGF-A is best known for its angiogenetic ability in embryogenesis and pathological conditions, [42] VEGF-B is important for the conservation and survival of pathologically formed blood vessels and in stressed conditions. [42, 43]

Increasing evidence show a specific role of VEGF-C and VEGF-R-3 in tumor lymphangiogenesis and lymphatic metastasis in multiple solid tumor types. [44–47] VEGFR-3 can be found on the endothelium of lymphatic vessels and some angiogenic tumor blood vessels. [48] Thus, this receptor is indispensable for both angiogenesis and lymphangiogenesis. [49] Recent experimental and clinical studies have implicated a positive association between VEGF-C expression, peritumoral lymphangiogenesis, and metastasis of malignant cells. [44, 50] It has been revealed that VEGF-C leads to an enlargement of peritumoral lymphatic vessels and increasing lymph flow, which facilitates the dissemination rate to lymph nodes and lymphatic intravasation. [44, 50] VEGF-C also contributes to tumor cell chemotaxis, which assists tumoral spread. [44, 45, 50] A positive correlation between the expression of VEGF-C and the extent of lymphatic metastasis has been found in breast, [17, 45] colorectal, [19] gastric, [25] thyroid, [22, 23] lung, [20, 45] and prostate [51] cancers. Like other malignancies, in the current study for the first time, significantly higher VEGF-C expression was detected in the metastatic melanomas. In similar studies VEGF-C expression was found significantly increased in metastatic melanoma compared to non-metastatic melanoma. [52–54] It has been also shown in the current study, that the low values of VEGF-C were associated with better disease-free and overall survival.

In the same way for VEGF-C, VEGF-D was found to be involved in lymphatic hyperplasia and metastatic spread to lymph nodes by activating VEGFR-2 and VEGFR-3. [44–47, 55, 56] VEGF-D overexpression has been also shown to lead tumor progression, enhanced metastasis and reduced survival, [57–59] as well as poorly differentiated histology and increased invasiveness. [60, 61] We have also detected significantly higher VEGFR-2 expression in the metastatic melanomas.

The current study is not without limitations. The donor blocks must be made using standard molds and cassettes as the instrument cannot adjust itself to various sizes. Additionally, donor blocks must exceed the minimal height (4 mm) to achieve optimal drilling. In some cases, this requires re-embedding of tissues. [32] Furthermore, sometimes the tissue cores do not attach properly to the slides and detach during the staining processes. However, ngTMA is a substantial improvement over conventional tissue microarraying techniques. It incorporates expertise in histology and ngTMA design with the flexibility of digital pathology and the precision of digital annotations with the speed and reliability of automated ngTMA construction. [32] In this method immunohistochemistry staining is performed only for one slide instead of hundred slides in the same condition with lower amount of needed antibodies and staining materials. This technique enables also image analysis for evaluation of protein and molecular biomarkers in a non-biased, fast, precise, fully-automated and quantitative manner.

In conclusion, since prognosis is a very crucial to prepare an individual therapy plan, imprecisions in guidelines lead to both over- und under-treatment. Despite extensive investigations to date, there is still a need for a non-invasive applicable prognostic marker to precisely identify the management strategy and follow the treatment efficacy of the high-risk malignant melanoma. The result of the current study declared that VEGF-C and VEGF-R2 expression as well as their extent might represent new prognostic markers in malignant melanoma. However, further prospective studies are warranted to test the accuracy and efficacy of these factors as new prognostic markers and possibly new individualized targets for antiangiogenic therapy in malignant melanoma. [62–64]

## Supporting information

S1 Fig ngTMA-block. (TIF)

S2 Fig Red staining was performed a cut from TMA-block stained using anti-VEGF-D antibody (R&D systems). The stained slide was scanned by panoramic Digital Slide Scanner (3DHISTECH). The unsuitable cores for analysis were marked as ’Missing data’ and excluded.
(TIF)

S3 Fig Loading plot showing the relationship between original variables and the first two components extracted by using principal component analysis (PCA). The angle between vectors is proportional to the degree of correlation between variables, while the length of vectors is proportional to the correlation between variables and PCA components.
(TIF)

S1 Table Staining protocols using BOND-III fully automated IHC stainer. (DOCX)

S2 Table Pearson’s correlations between each pair of scores. (DOCX)

S3 Table Distribution of study scores across demographics and clinical characteristics of patients with a diagnosis of MM. (DOCX)

S4 Table Distribution of study scores across demographics and clinical characteristics of patients with metastasis. (DOCX)

S5 Table Univariate analysis of the association between categorized scores and overall and disease-free patients’ survival. (DOCX)
