# Supplementary figures and images for: Correlation of Vascular Endothelial Growth Factor subtypes and their receptors with melanoma progression: A next-generation Tissue Microarray (ngTMA) automated analysis
Source: PLoS One. 2018 Nov 8;13(11):e0207019. doi: 10.1371/journal.pone.0207019 (PMC6224082; doi:10.1371/journal.pone.0207019)

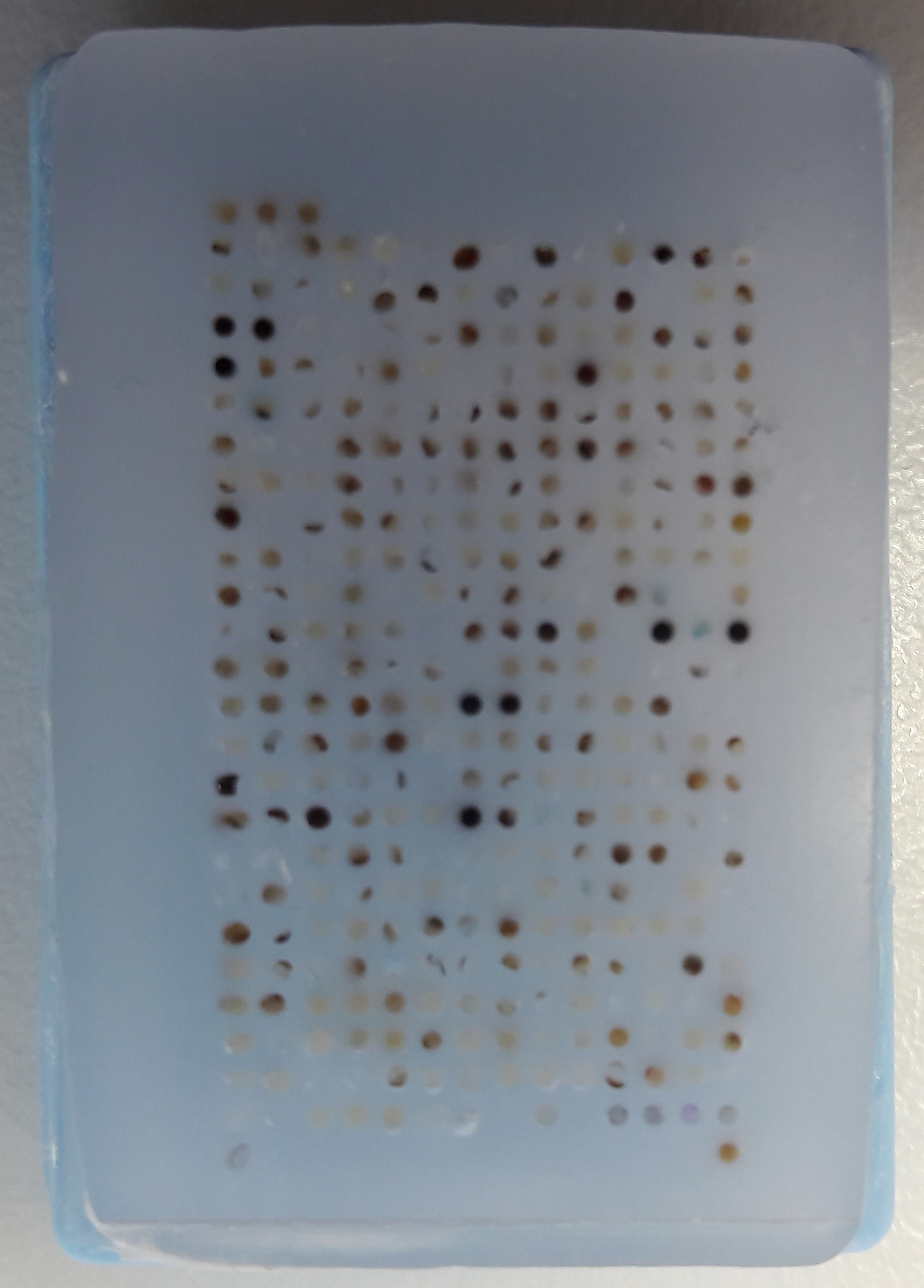

Supplement: S1 Fig — (TIF) [file pone.0207019.s001.tif]

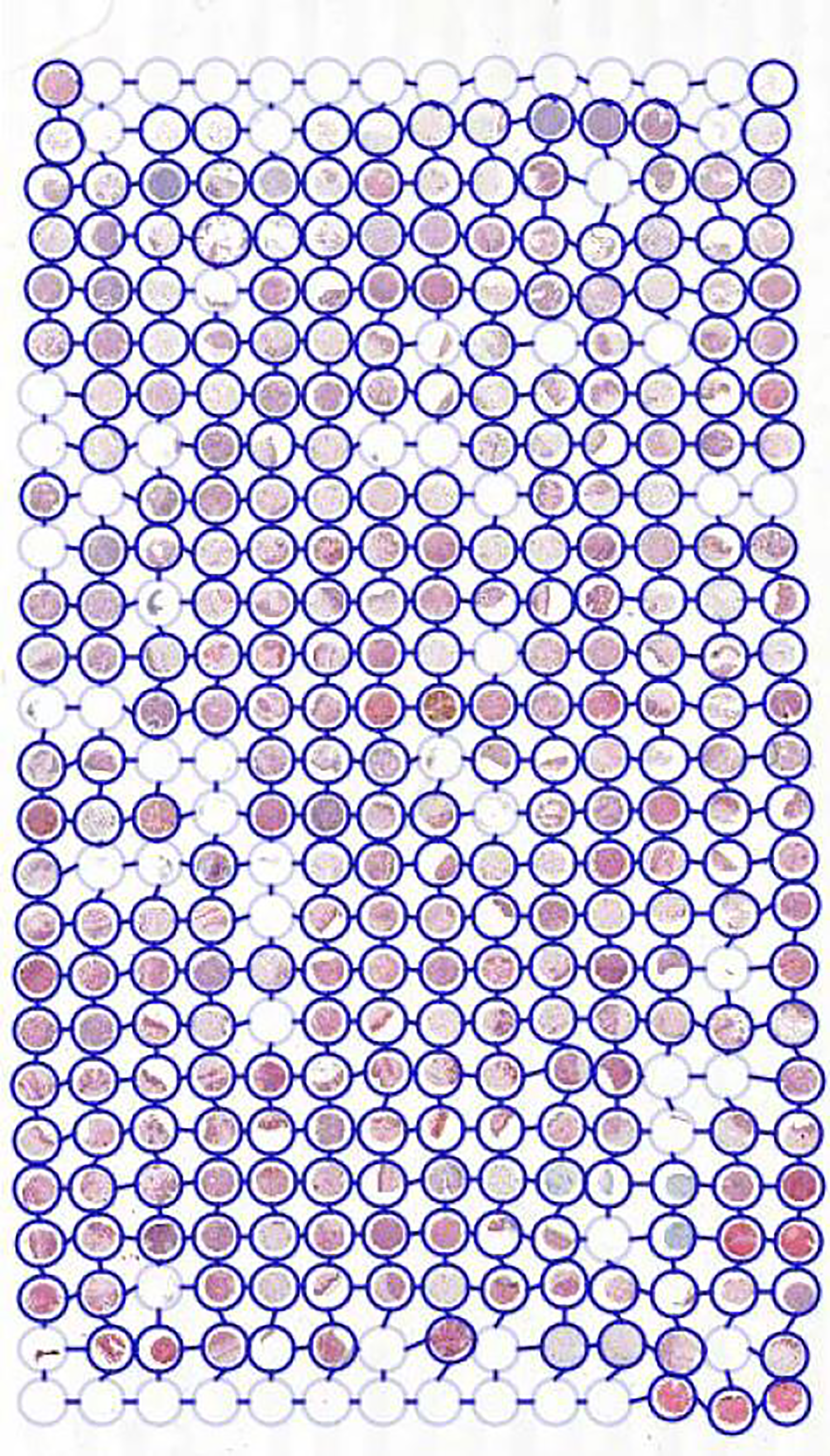

Supplement: S2 Fig — The stained slide was scanned by panoramic Digital Slide Scanner (3DHISTECH). The unsuitable cores for analysis were marked as ’Missing data’ and excluded. (TIF) [file pone.0207019.s002.tif]

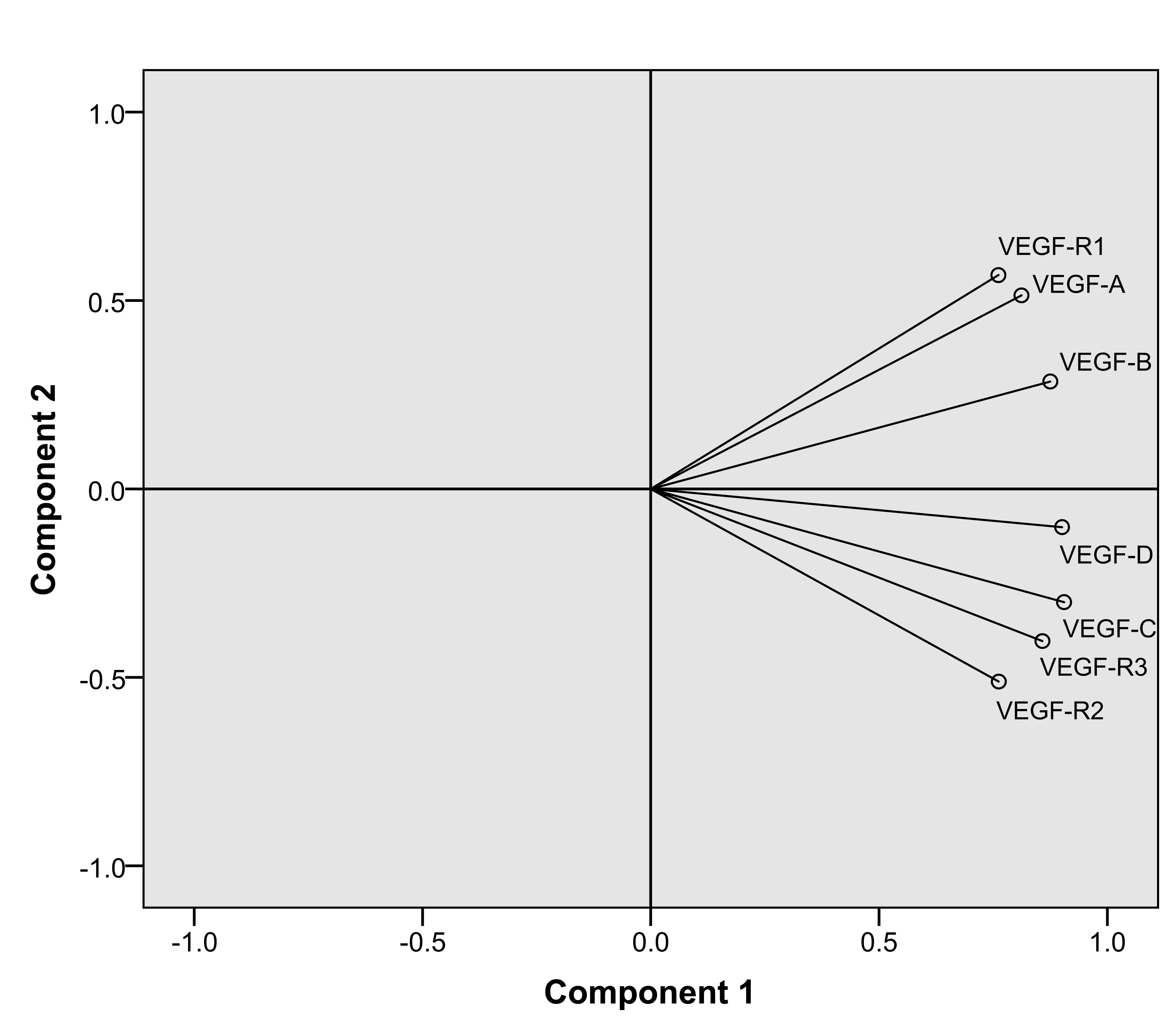

Supplement: S3 Fig — The angle between vectors is proportional to the degree of correlation between variables, while the length of vectors is proportional to the correlation between variables and PCA components. (TIF) [file pone.0207019.s003.tif]
