# Supplementary material for: Correlation of Vascular Endothelial Growth Factor subtypes and their receptors with melanoma progression: A next-generation Tissue Microarray (ngTMA) automated analysis
Source: PLoS One. 2018 Nov 8;13(11):e0207019. doi: 10.1371/journal.pone.0207019 (PMC6224082; doi:10.1371/journal.pone.0207019)
Supplement: S1 Table — (DOCX) [file pone.0207019.s004.docx]

**S 1 Table** – Staining protocols using BOND-III fully automated IHC stainer

| **Antibody** | **Company** | **Code** | **Staining Concentration** | **Pre-treatment und staining protocols** |
| --- | --- | --- | --- | --- |
| Anti-VEGFA | Abcom | ab155944 | 1:25 | H2(20)- Immuno |
| Anti-VEGFB | R&D | MAB751 | 1:50 | H2(20)- Immuno |
| Anti-VEGFC | Biorbyt | orb181623 | 1:200 | H2(20)- Immuno |
| Anti-VEGFD | R&D | MAB286 | 1:200 | H2(20)- Immuno |
| Anti-VEGFR1 | Biorbyt | orb27527 | 1:25 | H2 (30)- ICH L |
| Anti-VEGFR2 | R&D | MAB3571 | 1:25 | H2 (30)- ICH L |
| Anti-VEGFR3 | R&D | MAB3491 | 1:25 | H_1_(10)- Immuno |
