# Supplementary material for: Correlation of Vascular Endothelial Growth Factor subtypes and their receptors with melanoma progression: A next-generation Tissue Microarray (ngTMA) automated analysis
Source: PLoS One. 2018 Nov 8;13(11):e0207019. doi: 10.1371/journal.pone.0207019 (PMC6224082; doi:10.1371/journal.pone.0207019)
Supplement: S2 Table — (DOCX) [file pone.0207019.s005.docx]

**S2** **Table**– Pearson’s correlations between each pair of scores

|  | **VEGF-A** | **VEGF-B** | **VEGF-C** | **VEGF-D** | **VEGF-R1** | **VEGF-R2** | **VEGF-R3** |
| --- | --- | --- | --- | --- | --- | --- | --- |
| **VEGF-A** | 1 | 0.811 | 0.565 | 0.693 | 0.872 | 0.379 | 0.481 |
| **VEGF-B** | 0.811 | 1 | 0.683 | 0.716 | 0.780 | 0.539 | 0.622 |
| **VEGF-C** | 0.565 | 0.683 | 1 | 0.858 | 0.525 | 0.778 | 0.882 |
| **VEGF-D** | 0.693 | 0.716 | 0.858 | 1 | 0.571 | 0.655 | 0.772 |
| **VEGF-R1** | 0.872 | 0.780 | 0.525 | 0.571 | 1 | 0.320 | 0.451 |
| **VEGF-R2** | 0.379 | 0.539 | 0.778 | 0.655 | 0.320 | 1 | 0.812 |
| **VEGF-R3** | 0.481 | 0.622 | 0.882 | 0.772 | 0.451 | 0.812 | 1 |
