# Supplementary material for: Correlation of Vascular Endothelial Growth Factor subtypes and their receptors with melanoma progression: A next-generation Tissue Microarray (ngTMA) automated analysis
Source: PLoS One. 2018 Nov 8;13(11):e0207019. doi: 10.1371/journal.pone.0207019 (PMC6224082; doi:10.1371/journal.pone.0207019)
Supplement: S3 Table — (DOCX) [file pone.0207019.s006.docx]

**S3 Table** - Distribution of study scores across demographics and clinical characteristics of patients with a diagnosis of MM.

|  | | **VEGF-A** | **VEGF-B** | **VEGF-C** | **VEGF-D** | **VEGF-R1** | **VEGF-R2** | **VEGF-R3** | **P*** |
| --- | --- | --- | --- | --- | --- | --- | --- | --- | --- |
|  |  | **Mean, SD** | **Mean, SD** | **Mean, SD** | **Mean, SD** | **Mean, SD** | **Mean, SD** | **Mean, SD** |  |
| **Sex** | **F** | 1.31, 1.26 | 1.76, 0.83 | 5.18, 3.19 | 3.87, 2.25 | 0.66, 0.63 | 12.86, 4.92 | 8.91, 5.02 | 0.551 |
|  | **M** | 1.33, 1.36 | 1.90, 1.75 | 6.23, 5.75 | 4.58, 3.71 | 0.80, 1.33 | 12.98, 7.18 | 9.82, 7.62 |  |
| **Age (years)** | **< 50** | 1.11, 0.53 | 1.57, 0.86 | 5.72, 5.56 | 4.16, 3.51 | 0.53, 0.37 | 12.09, 6.29 | 9.12, 7.03 | 0.819 |
|  | **50 - 64** | 1.69, 2.09 | 2.05, 1.98 | 5.65, 4.18 | 4.41, 3.12 | 0.97, 1.75 | 12.70, 6.05 | 9.16, 6.47 |  |
|  | **65+** | 1.14, 0.67 | 1.82, 1.19 | 5.81, 4.97 | 4.17, 3.08 | 0.67, 0.57 | 13.41, 6.63 | 9.63, 6.70 |  |
| **Tumour type** | **NM** | 1.36, 0.81 | 2.14, 1.16 | 10.35, 7.45 | 6.61, 4.75 | 0.92, 0.76 | 17.45, 8.50 | 15.00, 9.56 | <0.001 |
|  | **SSM** | 1.22, 1.08 | 1.61, 0.90 | 4.60, 3.00 | 3.66, 2.32 | 0.61, 0.54 | 11.73, 5.26 | 8.07, 4.84 |  |
|  | **Other** | 1.55, 2.13 | 2.22, 2.56 | 4.91, 4.07 | 3.86, 2.82 | 0.95, 2.14 | 11.96, 5.23 | 8.02, 5.72 |  |
| **Primary tumour** | **Head and Neck** | 0.96, 0.64 | 1.33, 0.97 | 3.12, 2.26 | 2.69, 1.62 | 0.49, 0.44 | 10.66, 5.51 | 5.95, 5.09 | 0.578 |
| **location** | **Trunk** | 1.52, 1.73 | 2.04, 1.77 | 6.06, 5.03 | 4.31, 3.37 | 0.95, 1.46 | 12.51, 6.26 | 9.61, 6.60 |  |
|  | **Upper Ext.** | 1.22, 0.46 | 1.72, 0.74 | 5.99, 3.59 | 4.63, 2.21 | 0.60, 0.27 | 15.47, 6.14 | 10.41, 6.21 |  |
|  | **Lower Ext.** | 1.05, 0.55 | 1.75, 1.03 | 6.82, 6.10 | 4.77, 3.93 | 0.49, 0.22 | 13.78, 7.14 | 10.70, 7.91 |  |
| **Ulceration** | **No** | 1.35, 1.41 | 1.90, 1.53 | 5.85, 5.05 | 4.27, 3.26 | 0.78, 1.17 | 12.91, 6.37 | 9.26, 6.67 | 0.54 |
|  | **Yes** | 1.18, 0.51 | 1.57, 0.59 | 5.68, 3.80 | 4.45, 2.92 | 0.55, 0.30 | 12.84, 6.69 | 10.49, 6.98 |  |
| **Breslow’s thickness** | **Thin** | 1.04, 0.52 | 1.38, 0.65 | 3.81, 2.17 | 3.03, 1.82 | 0.51, 0.29 | 10.35, 4.32 | 6.69, 3.89 | 0.009 |
|  | **Intermediate** | 1.76, 1.91 | 2.51, 1.96 | 8.70, 6.16 | 6.22, 3.79 | 1.10, 1.63 | 16.49, 7.00 | 13.33, 7.93 |  |
|  | **Thick** | 0.70, 0.12 | 1.44, 0.44 | 3.57, 2.25 | 2.09, 0.68 | 0.34, 0.14 | 11.80, 6.54 | 7.42, 4.42 |  |
| **SLNB** | **Negative** | 1.38, 1.23 | 1.89, 1.02 | 6.83, 5.34 | 5.04, 3.66 | 0.65, 0.61 | 14.43, 6.50 | 10.96, 6.56 | 0.127 |
| **findings** | **Positive** | 2.08, 2.92 | 3.24, 3.39 | 8.23, 6.62 | 5.73, 4.14 | 1.80, 2.93 | 15.24, 6.82 | 12.83, 9.55 |  |

NM: Nodular melanoma, MM: malignant melanoma, P: p-value, SD: standard deviation, SLNB: Sentinel lymph node biopsy , SSM: Superficial spreading melanoma

* One-way MANOVA overall p-value.
