# Supplementary material for: Correlation of Vascular Endothelial Growth Factor subtypes and their receptors with melanoma progression: A next-generation Tissue Microarray (ngTMA) automated analysis
Source: PLoS One. 2018 Nov 8;13(11):e0207019. doi: 10.1371/journal.pone.0207019 (PMC6224082; doi:10.1371/journal.pone.0207019)
Supplement: S4 Table — (DOCX) [file pone.0207019.s007.docx]

**S4 Table**- Distribution of study scores across demographics and clinical characteristics of patients with metastasis.

|  | | **VEGF-A** | **VEGF-B** | **VEGF-C** | **VEGF-D** | **VEGF-R1** | **VEGF-R2** | **VEGF-R3** | **P*** |
| --- | --- | --- | --- | --- | --- | --- | --- | --- | --- |
|  |  | **Mean, SD** | **Mean, SD** | **Mean, SD** | **Mean, SD** | **Mean, SD** | **Mean, SD** | **Mean, SD** |  |
| **Sex** | **F** | 1.46, 1.12 | 2.23, 1.61 | 7.03, 3.91 | 4.85, 2.24 | 0.7, 0.59 | 14.55, 7.48 | 10.54, 5.49 | 0.022 |
|  | **M** | 1.10, 0.67 | 1.78, 0.95 | 6.83, 4.7 | 3.68, 2.30 | 0.64, 0.58 | 15.58, 6.51 | 12.19, 6.84 |  |
| **Age (years)** | **< 50** | 1.42, 0.80 | 2.09, 1.18 | 7.72, 4.51 | 5.32, 2.84 | 0.96, 1.05 | 17.65, 7.16 | 13.1, 6.93 | 0.145 |
|  | **50 - 64** | 1.43, 1.39 | 2.45, 1.99 | 9.05, 6.62 | 5.33, 3.13 | 0.74, 0.71 | 18.12, 8.57 | 13.85, 9.8 |  |
|  | **65+** | 1.07, 0.61 | 1.69, 0.81 | 5.84, 3.03 | 3.30, 1.45 | 0.53, 0.27 | 13.46, 5.69 | 10.48, 4.39 |  |
| **Tumor type** | **NM** | 1.20, 0.69 | 1.79, 0.96 | 6.42, 3.78 | 3.74, 2.10 | 0.59, 0.25 | 14.36, 7.25 | 10.92, 5.88 | 0.245 |
|  | **SSM** | 1.10, 0.56 | 1.88, 1.06 | 8.72, 6.45 | 4.45, 2.67 | 0.51, 0.34 | 18.38, 5.57 | 14.02, 7.99 |  |
|  | **Other** | 1.06, 0.73 | 1.84, 0.92 | 6.01, 3.62 | 3.97, 2.25 | 0.7, 0.81 | 13.01, 6.02 | 10.39, 5.8 |  |
| **Primary tumor** | **Head and Neck** | 0.93, 0.50 | 1.45, 0.69 | 5.09, 2.44 | 2.73, 1.45 | 0.49, 0.27 | 11.69, 5.59 | 9.17, 4.89 | 0.005 |
| **location** | **Trunk** | 1.24, 0.47 | 1.89, 0.67 | 7.39, 3.55 | 4.85, 1.87 | 0.57, 0.24 | 18.05, 7.63 | 12.39, 6.23 |  |
|  | **Upper Ext.** | 0.93, 0.55 | 1.47, 0.61 | 6.23, 4.01 | 2.71, 1.03 | 0.54, 0.32 | 14.17, 5.07 | 11.92, 4.93 |  |
|  | **Lower Ext.** | 1.74, 1.28 | 2.84, 1.78 | 8.97, 6.24 | 5.90, 2.64 | 0.97, 0.95 | 18.02, 6.84 | 14.18, 8.11 |  |
| **Ulceration** | **No** | 1.12, 0.60 | 1.96, 0.94 | 8.87, 5.11 | 4.62, 2.58 | 0.56, 0.32 | 18.54, 5.99 | 14.59, 7.01 | 0.486 |
|  | **Yes** | 1.18, 0.63 | 1.77, 0.85 | 6.09, 4.01 | 3.93, 2.16 | 0.7, 0.71 | 14.45, 6.63 | 10.25, 5.49 |  |
| **Breslow’s thickness** | **Thin** | 0.64, 0.29 | 1.18, 0.62 | 4.04, 2.25 | 2.46, 1.43 | 0.38, 0.2 | 9.62, 4.94 | 7.1, 4.33 | 0.539 |
|  | **Intermediate** | 1.23, 0.69 | 2.06, 1.00 | 7.58, 4.78 | 4.47, 2.17 | 0.58, 0.3 | 17.05, 6.91 | 12.41, 6.52 |  |
|  | **Thick** | 1.25, 0.63 | 1.79, 0.88 | 6.99, 4.35 | 3.90, 2.50 | 0.77, 0.78 | 13.98, 6.01 | 11.12, 6.25 |  |
| **SLNB** | **Negative** | 1.16, 0.55 | 1.87, 0.71 | 7.77, 4.99 | 3.85, 1.89 | 0.66, 0.27 | 15.85, 7.09 | 12.27, 7.63 | 0.518 |
| **findings** | **Positive** | 1.31, 1.02 | 2.01, 1.37 | 7.15, 4.76 | 5.07, 3.03 | 0.75, 0.99 | 16.34, 7.58 | 12.7, 7.69 |  |

NM: Nodular melanoma, MM: malignant melanoma, P: p-value, SD: standard deviation, SLNB : Sentinel lymph node biopsy , SSM: Superficial spreading melanoma* One-way MANOVA overall p-value.
