# Supplementary material for: Correlation of Vascular Endothelial Growth Factor subtypes and their receptors with melanoma progression: A next-generation Tissue Microarray (ngTMA) automated analysis
Source: PLoS One. 2018 Nov 8;13(11):e0207019. doi: 10.1371/journal.pone.0207019 (PMC6224082; doi:10.1371/journal.pone.0207019)
Supplement: S5 Table — (DOCX) [file pone.0207019.s008.docx]

**S5 Table**- Univariate analysis of the association between categorized scores and overall and disease-free patients’ survival.

|  | | **Overall survival**  **(at 15 years)** | | | **Disease-free survival**  **(at 15 years)** | | |
| --- | --- | --- | --- | --- | --- | --- | --- |
|  |  | **Cut-off*** | **N, %** | **P**** | **Cut-off*** | **N, %** | **P**** |
| **VEGF-A** | **low** | <1.5 | 92, 67.5 | 0.242 | <1.5 | 91, 51.7 | 0.137 |
|  | **high** | 1.5+ | 40, 66.6 |  | 1.5+ | 40, 47.0 |  |
| **VEGF-B** | **low** | <2.2 | 96, 77.9 | 0.217 | <2.1 | 93, 61.2 | 0.067 |
|  | **high** | 2.2+ | 36, 51.6 |  | 2.1+ | 38, 28.0 |  |
| **VEGF-C** | **low** | <5.7 | 76, 80.9 | 0.007 | <7.6 | 96, 64.3 | <0.001 |
|  | **high** | 5.7+ | 56, 50.6 |  | 7.6+ | 35, 21.7 |  |
| **VEGF-D** | **low** | <4.2 | 79, 76.2 | 0.104 | <4.2 | 78, 60.5 | 0.050 |
|  | **high** | 4.2+ | 53, 55.0 |  | 4.2+ | 53, 34.7 |  |
| **VEGF-R1** | **low** | <0.9 | 110, 69.1 | 0.042 | <0.9 | 109, 53.0 | 0.044 |
|  | **high** | 0.9+ | 22, 52.0 |  | 0.9+ | 22, 31.1 |  |
| **VEGF-R2** | **low** | <12.9 | 64, 80.1 | 0.023 | <16.5 | 88, 63.9 | <0.001 |
|  | **high** | 12.9+ | 68, 57.3 |  | 16.5+ | 43, 19.4 |  |
| **VEGF-R3** | **low** | <12.7 | 98, 74.9 | 0.026 | <8.5 | 65, 71.5 | 0.001 |
|  | **high** | 12.7+ | 34, 52.7 |  | 8.5+ | 66, 31.6 |  |

* Optimal cut-off based on significance of correlation with survival outcomes

** Log-rank test
